# Estrogen receptor α phosphorylated at Ser216 confers inflammatory function to mouse microglia

**DOI:** 10.1186/s12964-020-00578-x

**Published:** 2020-07-29

**Authors:** Sawako Shindo, Shih-Heng Chen, Saki Gotoh, Kosuke Yokobori, Hao Hu, Manas Ray, Rick Moore, Kiyoshi Nagata, Jennifer Martinez, Jau-Shyong Hong, Masahiko Negishi

**Affiliations:** 1 Pharmacogenetics, Reproductive and Developmental Biology Laboratory, National Institute of Environmental Health Sciences, National Institutes of Health, Research Triangle Park, North Carolina 27709 USA; 2 Departments of Environmental Health, Faculty of Pharmaceutical Sciences, Tohoku Medical and Pharmaceutical University, Sendai, 981-8558 Japan; 3 Neurobiology Laboratory, National Institute of Environmental Health Sciences, National Institutes of Health, Research Triangle Park, North Carolina 27709 USA; 4 Knockout Mouse Core, National Institute of Environmental Health Sciences, National Institutes of Health, Research Triangle Park, North Carolina 27709 USA; 5 Immunity, Inflammation and Disease Laboratory, National Institute of Environmental Health Sciences, National Institutes of Health, Research Triangle Park, North Carolina 27709 USA

**Keywords:** Nuclear receptor, Estrogen receptor, Brain, Microglia, Inflammation, Phosphorylation

## Abstract

**Background:**

Estrogen receptor α (ERα) has been suggested to regulate anti-inflammatory signaling in brain microglia, the only resident immune cells in the brain. ERα conserves the phosphorylation motif at Ser216 within the DNA binding domain. Previously, Ser216 was found to be phosphorylated in neutrophils infiltrating into the mouse uterus and to enable ERα to regulate migration. Given the implication of this phosphorylation in immune regulation, ERα was examined in mouse microglia to determine if Ser216 is phosphorylated and regulates microglia’s inflammation. It was found that Ser216 was constitutively phosphorylated in microglia and demonstrated that in the absence of phosphorylated ERα in ERα KI brains microglia inflamed, confirming that phosphorylation confers ERα with anti-inflammatory capability. ERα KI mice were obese and weakened motor ability.

**Methods:**

Mixed glia cells were prepared from brains of 2-days-old neonates and cultured to mature and isolate microglia. An antibody against an anti-phospho-S216 peptide of ERα (αP-S216) was used to detect phosphorylated ERα in double immunofluorescence staining with ERα antibodies and a microglia maker Iba-1 antibody. A knock-in (KI) mouse line bearing the phosphorylation-blocked ERα S216A mutation (ERα KI) was generated to examine inflammation-regulating functions of phosphorylated ERα in microglia. RT-PCR, antibody array, ELISA and FACS assays were employed to measure expressions of pro- or anti-inflammatory cytokines at their mRNA and protein levels. Rotarod tests were performed to examine motor connection ability.

**Results:**

Double immune staining of mixed glia cells showed that ERα is phosphorylated at Ser216 in microglia, but not astrocytes. Immunohistochemistry with an anti-Iba-1 antibody showed that microglia cells were swollen and shortened branches in the substantial nigra (SN) of ERα KI brains, indicating the spontaneous activation of microglia as observed with those of lipopolysaccharide (LPS)-treated ERα WT brains. Pro-inflammatory cytokines were up-regulated in the brain of ERα KI brains as well as cultured microglia, whereas anti-inflammatory cytokines were down-regulated. FACS analysis showed that the number of IL-6 producing and apoptotic microglia increased in those prepared from ERα KI brains. Times of ERα KI mice on rod were shortened in Rotarod tests.

**Conclusions:**

Blocking of Ser216 phosphorylation aggravated microglia activation and inflammation of mouse brain, thus confirming that phosphorylated ERα exerts anti-inflammatory functions. ERα KI mice enable us to further investigate the mechanism by which phosphorylated ERα regulates brain immunity and inflammation and brain diseases.

Video abstract

## Background

Estrogen and estrogen receptor α (ERα) are known to regulate anti-inflammatory signaling in the brain [1] and are directly involving in the pathogenesis of neurodegeneration and other inflammation-related brain diseases [2]. Microglia are the resident macrophages in the brain, responsible for the control of neuroinflammation [3]. Since ERα is ubiquitously expressed in the brain, the signaling that specifically regulates ERα in microglia remains uninvestigated. Moreover, a proper animal model that enables us to perform targeted examinations to microglia in brain does not currently exist. Here we have now found that ERα is phosphorylated at Ser216 in microglia and generated an ERαS216A knock-in (*Esr1*^*S216A*^) mouse line to investigate whether this phosphorylation enables ERα to regulate inflammation of microglia. ERα’s Ser216 (Ser212 in human ERα) is present within the DNA binding domain (DBD) and conserved a phosphorylation motif in 41 out of 46 total mouse nuclear receptors (Additional file 1: Figure S1) as well as in the corresponding human nuclear receptors [4]. These extremely high cross-species conservations strongly suggest critical regulatory functions this motif may plays for nuclear receptor actions. In fact, In addition to Ser216 of ERα, the corresponding residues of four other nuclear receptors have been reported to be phosphorylated in mouse tissues in vivo and to confer specific function to them, which include Thr38, Ser100, Ser154 and Thr167 of constitute active/androstane receptor (CAR), retinoid-related orphan receptors α (RORα), farnesoid X receptor (FXR) and retinoid X receptor α (RXRα), respectively [4–8]. For example, phosphorylation of Thr38 presses the constitutive activity of CAR, providing it with the response ability to its activator [5, 6]. RXRα was found to be phosphorylated at Thr167 in mouse adipose tissues in response to fasting. We generated RXRα T167A knock-in mouse and demonstrated that this phosphorylation regulates blood glucose levels by altering energy metabolism in adipose tissues [8]. These observations confirmed that phosphorylation of this conserved motif can be a common regulatory factor of nuclear receptors. Thus, phosphorylation of Ser216 presents an experimental basis to examine ERα in this context. Post-translational modifications are known to be important for protein activities. We previously showed that human ERα S212 mutants regulated the different group of genes form those regulated when they were overexpressed in Huh-7 cells [9]. Moreover, it was revealed that serine 216 of ERα was phosphorylated in vivo in neutrophils infiltrating the mouse uterus using a specific phosphorylated ERα recognition antibody [10]. With respect to phosphorylation of ERα, various residues were reported such as Ser118 and Ser167 in uterine fibroids or breast cancer cells [11, 12], although phosphorylation has not been observed with endogenous ERα in tissues in vivo. On the other hand, Ser216 enabled us to investigate the in vivo function of phosphorylated ERα.

This manuscript analyzed inflammation of microglia of the ERα KI brain in comparison with that of ERα WT brains. Microglia in the brain and, subsequently, glia cells cultured from the brains of 2-day-old neonates were subjected to investigations by using immunohistochemistry, real-time PCR, ELISA, cytokine arrays, FACS and Western blots. With experimental observations obtained we will discuss the anti-inflammatory and anti-apoptotic functions of phosphorylated ERα in microglia. This ERα KI (*Esr1*^*S216A*^) mouse is the first KI at a potential phosphorylation motif that shows a physiological phenotype and can be used as an animal model for the study of physiological functions of phosphorylated ERα and their molecular mechanism as well as for drug discovery and development targeting microglia.

## Results

### ERα phosphorylated at Ser216 in microglia

**Fig. 1:**
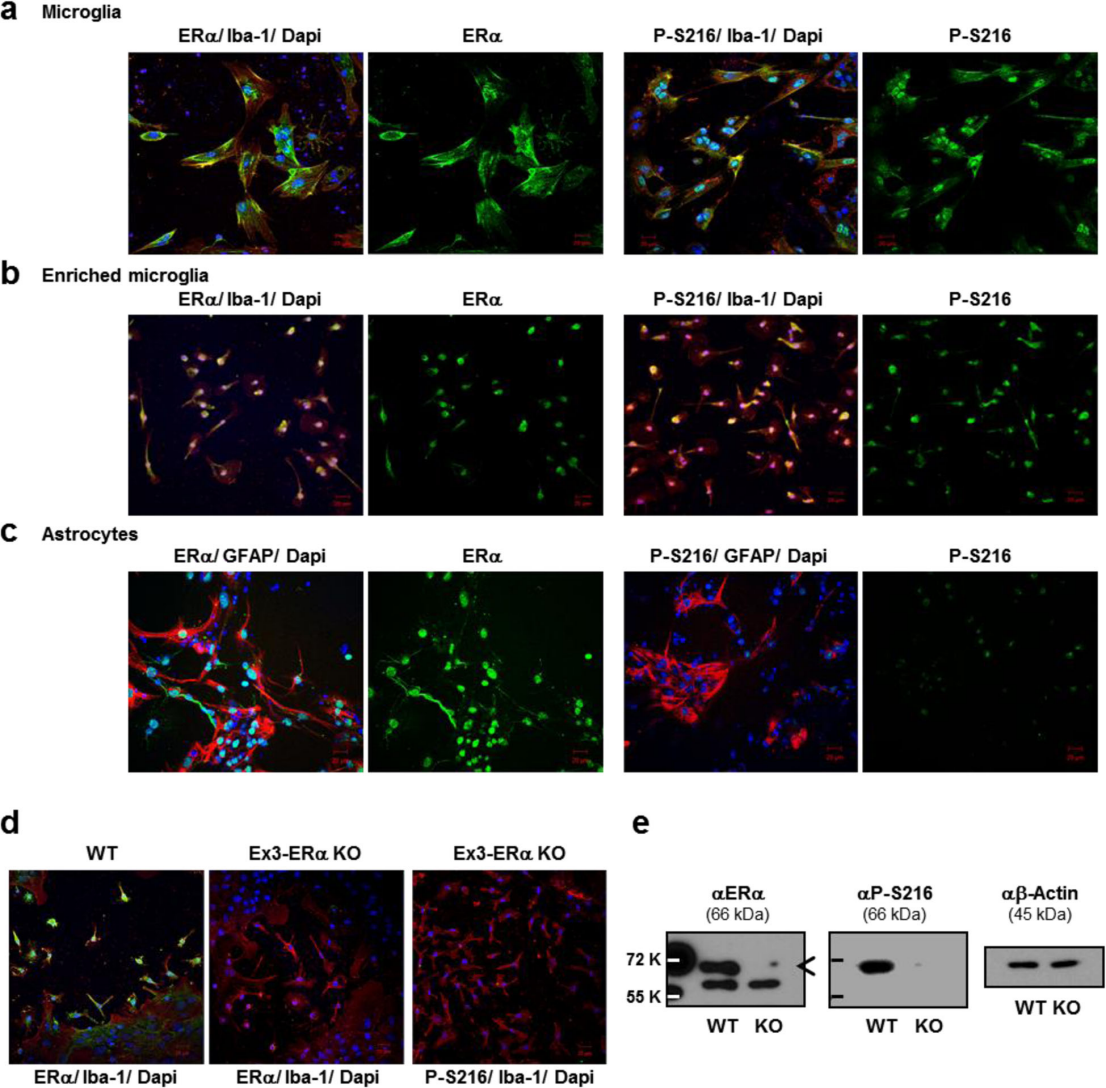
ERα phosphorylated at Ser216 in mouse brain microglia. **a** Microglia. Mixed glia cells were prepared, cultured for 4-days and double-stained with either an anti-ERα (in green) or an anti-P-S216 (in green) antibody and an anti-Iba-1 (in red) antibody for microglia marker and visualized as described in the Methods section. Nuclei were stained by DAPI in blue. **b** Enriched microglia. Microglia was enriched from glia cells as described in the Methods section for double staining. **c** Astrocytes. Mixed glia cells were double-stained with either an anti-ERα (in green) or an anti-P-S216 (in green) antibody and an anti-GFAP (in red) antibody for astrocyte marker and were visualized as described in the Methods section. Nuclei were stained by DAPI in blue. **d** Glia cells obtained from 2-days-old neonates of Ex3-ERα KO and wild type (WT) mice were cultured and double-stained by an anti-ERα or an anti-P-Ser-216 (P-S216) (in green) and an anti-Iba-1 (in red) antibodies and visualized as described in the Methods section. Nuclei were stained by DAPI in blue. **e** Whole lysates prepared from enriched microglia were subjected to Western blots by an anti-ERα or an anti- P-S216 antibody. An an anti-β-Actin antibody was utilized to verify equal amounts of proteins in an each well

Mixed glia cells were isolated from brains of 2-day-old neonates and cultured for 2 weeks to mature microglia prior to immunofluorescence staining. For microglia, these glia cells were double stained with an anti-ERα or P-S216 peptide antibody (in green) with an anti-Iba-1 antibody (in red) (Fig. 1a). In microglia, both ERα antibodies stained the cytoplasm and nucleus, whereas a phosphorylated ERα antibody appeared to strongly stain the nucleus. Staining of ERα by an anti-P-S216 peptide antibody was further confirmed with enriched mature microglia from glia cultures (Fig. 1b). In these cells, no staining differences between two antibodies were observed. For astrocytes, glia cells were co-stained by an anti-ERα or P-S216 peptide antibody (in green) with an anti-GFAP antibody (in red) (Fig. 1c). Anti-ERα antibody strongly stained astrocytes, while the P-S216 peptide antibody barely stained these cells. Obtained observations indicate that ERα is phosphorylated at serine 216 in microglia and were expressed in the nucleus as well as in the extra-nuclear region. ERα KO mice were further examined ERα in brain microglia. First, immunostaining was performed to show the absence of ERα in microglia (Fig. 1d). Whole extracts from enriched microglia were subjected to Western blot analysis. Both an anti-ERα and P-S216 antibodies detected ERα band only in the extracts from ERα WT microglia (Fig. 1e).

### ERα S216A KI (*Esr1*^*S216A*^) mice

**Fig. 2:**
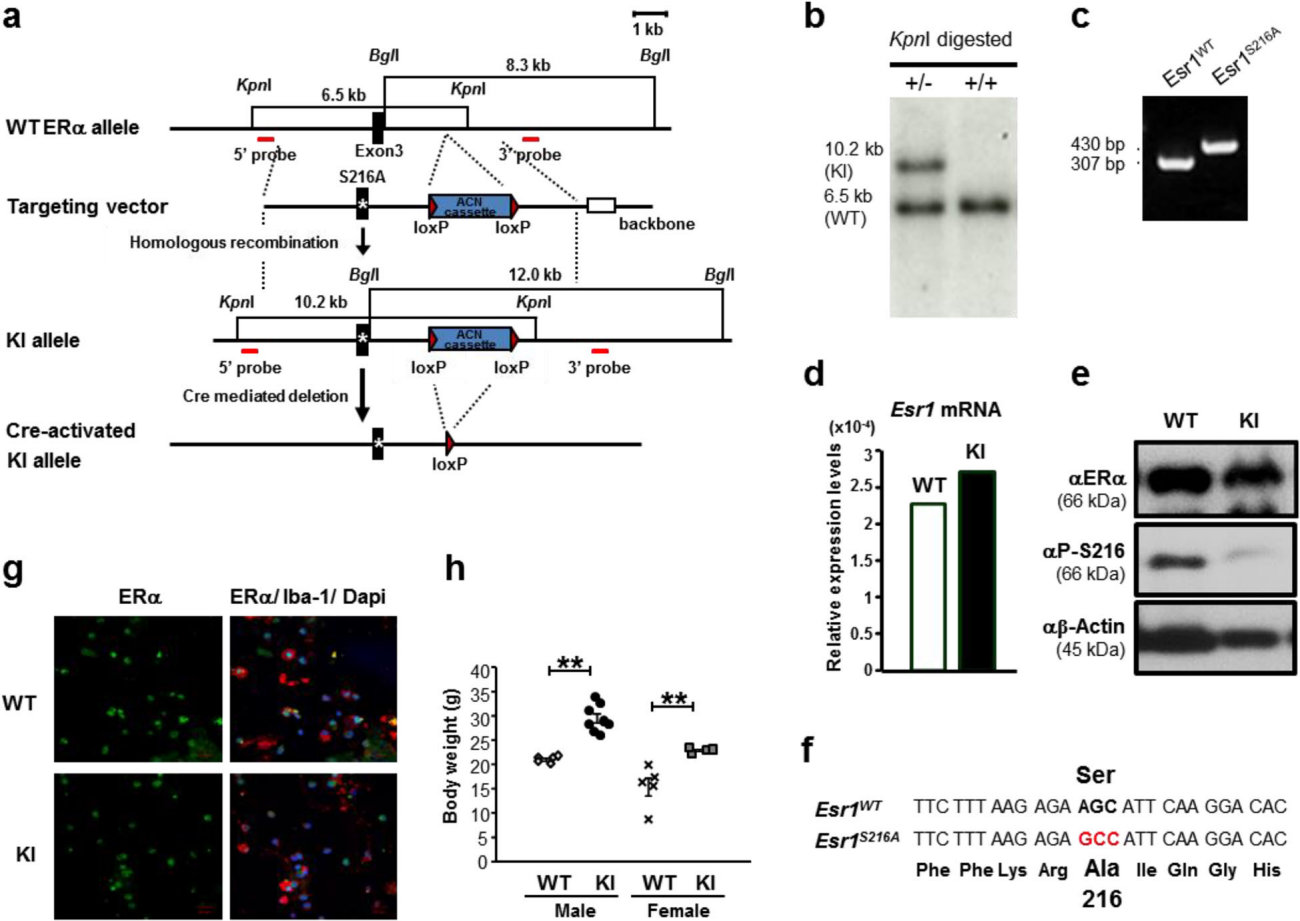
Generation and phenotypes of ERα S216A KI (*Esr1*^*S216A*^) mice. **a** Map of strategy and Knock-in process. **b** Southern blot analyses were performed to identify the appropriately targeted allele. Genomic DNAs from mouse ear biopsies were digested with KpnI and BglI. KpnI-digestion generated 10.2- and 6.5-kb bands for knock-in (KI) and wild type (WT), respectively, detected by using the 5′-genomic probe. **c** Genotyping by PCR. Genomic DNA was isolated from mouse ear biopsies. PCR primers were used to amplify across the region in which the one remaining loxP site was inserted. This gave a band of 430 bp for a knock-in allele and 307 bp for a wild-type allele. Self-excision of the ACN cassette in the mutant allele was confirmed. **d** Expression of ERα mRNA. RNAs were prepared from WT and KI uteri and subjected to real time PCR analysis. These are averages from duplicates of two independent samples. **e** Conformation of ERα protein expression in WT and KI uteri. Whole extracts (15 μg proteins) prepared from WT and KI uteri were subjected to Western blots by an anti-ERα, an anti-P-Ser-216 (P-S216) or an anti-β-Actin antibody. **f** Sequence conformation of the mutation. cDNAs were synthesized from WT and KI uteri and sequenced. **g** ERα expression in ERα KI microglia. Accumulated microglia in the glia cells, which were prepared from brains of 2 days-old neonates of ERα KI males, were subjected to fluorescence staining with an anti-ERα (in green) antibody or double staining with an anti-Iba-1 (in red) antibody. DAPI stained nuclei in blue. **h** Obese phenotype of ERα S216A KI mice. Six-month-old mice were weighed; 5 males and 5 females of WT and 8 males and 4 females of KI mice. One Way ANOVA plus post hoc test with Tukey-Kramer’s multiple comparisons test (Version 5.0, Stat view-j) was used for statistical analysis. Values are presented as means ± S.E.. **, *p* < 0.01

Utilizing ACN cassette, a single mutation of serine 216 to alanine was introduced in the Esr1 gene (Fig. 2a). The mutation was verified by Southern hybridization (Fig. 2b) and PCR amplification (Fig. 2c). ERα mRNA and protein were equally expressed in the uterus of ERα WT and ERα KI mice (Fig. 2d and e) and the sequences of cDNAs confirmed the mutation (Fig. 2f). Moreover, co-immunostaining of glia cells with an anti-ERα and an anti-Iba-1 antibodies demonstrated the presence of ERα in microglia prepared from ERα KI as observed as with ERα WT mice (Fig. 2g). ERα KI mice were fertile; the pups were born normally in either ERα KI females or males. However, both male and female ERα KI mice developed obesity. At 6-month-old mice ERα KI mice were about 40% over-weight compared to WT mice (Fig. 2h).

### Microglia in adult ERα KI brains

**Fig. 3:**
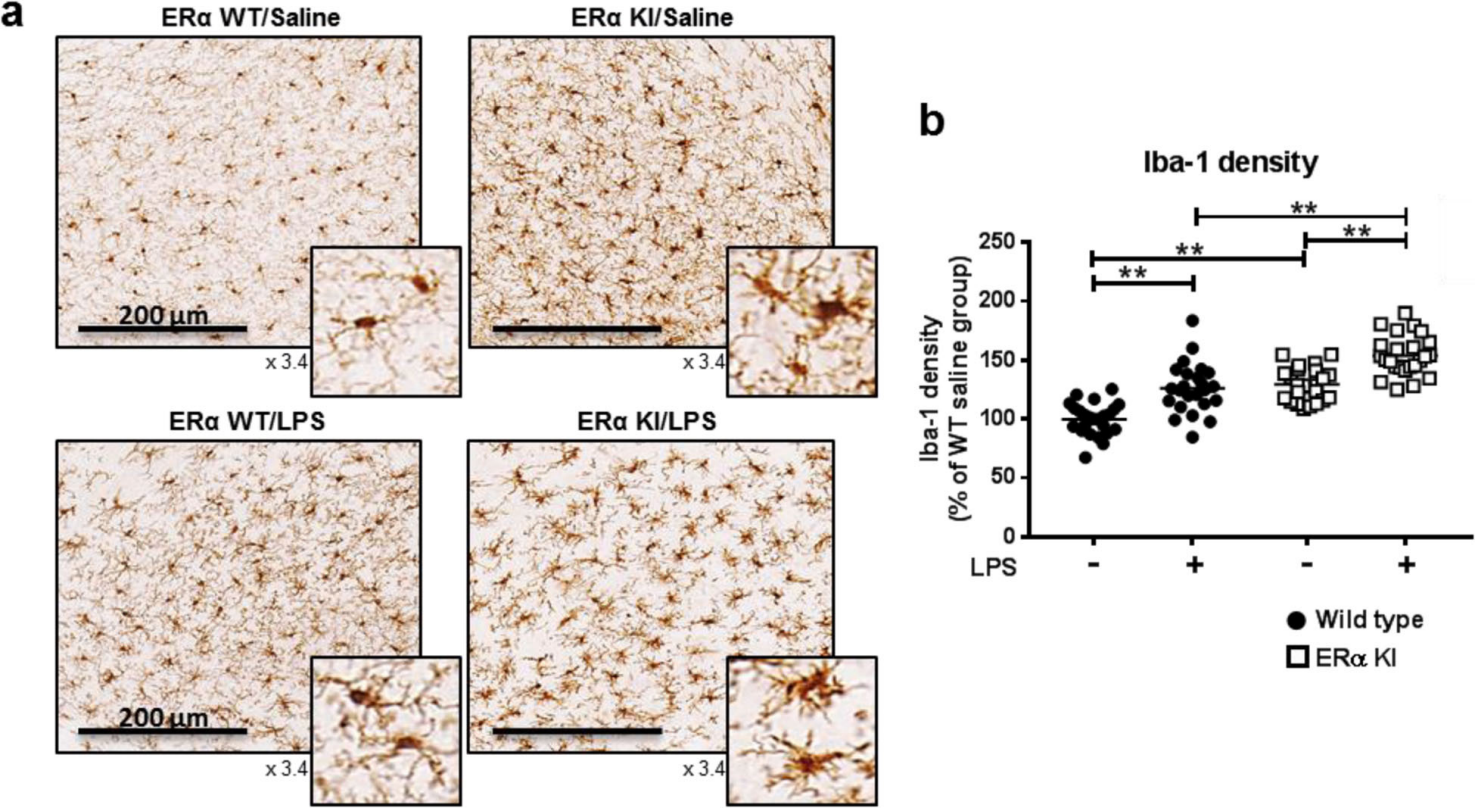
Microglia in substantia nigra of adult brains. **a** Saline or LPS (5 mg/kg, ip., 2 h)-treated brains. Brains were removed from 6-month-old ERα WT and KI males, from which frozen sections were prepared for immunostaining with an anti Iba-1 antibody. The brain region showing the microglia morphological changes is substantia nigra (SN). **b** Three brains were used for each group. Four sections from each brain were stained for analysis. Stained microglia were scanned to semi-quantitate microglia activation. Values are presented as means ± S.E., by taking values of ERα WT as 100%. Statistical analysis was conducted with One Way ANOVA plus post hoc test with Bonferroni’s multiple comparisons test (Version 7.00, GraphPad, San Diego, CA). **, p < 0.01

**Fig. 4:**
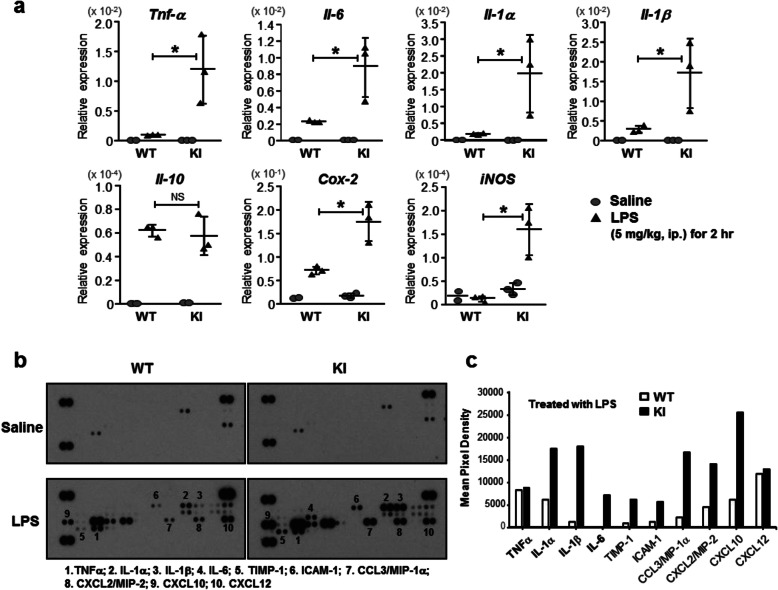
Expression of inflammatory factors in adult brains. **a** qRT-PCR. Adult WT or KI male mice were treated with intraperitoneally injected LPS (5 mg/kg, ip.) or saline for each group for 2 h, from the whole brains of which RNAs were prepared for subsequent analysis. Assay was duplicated for each RNA sample. With 6 RT-PCR data for either WT or KI, Two Way ANOVA plus post hoc test with Bonferroni’s multiple comparisons test (Version 5.0, Stat view-j) was used for statistical analysis. Values are presented as means ± S.D.. *, *p* < 0.05, *n* = 3. **b** Cytokine antibody arrays. From the same brains used in (**a**), whole extracts were prepared, 300 μg proteins of which were subjected to analysis. **c** Mean Pixel Densities were obtained from each spot using Image J soft

Although microglia are present throughout mouse brain, its distribution is much denser in specific regions such as olfactory telencephalon and substantia nigra (SN) reticulata [13]. Brain sections were prepared from saline- or LPS-treated adult ERα WT and KI males and stained with an anti-Iba-1 antibody (Fig. 3a). Intensities of Iba-1 staining are known to increase as microglia become more activated [14]. Those intensities were higher in saline-treated ERα KI compared with those in ERα WT. Moreover, increases in the staining intensity by LPS treatment were 20% higher in ERα KI than in ERα WT mice (Fig. 3b). In addition, microglia developed morphological changes such as hypertrophic cells with pseudopodia in ERα KI mice (Fig. 3a). In the ERα WT /saline group, the microglia are small, spherical and rod-shaped cells, typifying morphology of resting microglia. On the other hand, in ERα KI/saline brains, microglia are swelled, shortened and thickened, representing an active state, similar to those of ERα WT/LPS or ERα KI/LPS brains. These pictures represent a distinct state of microglia in ERα WT and KI mice. Microglial activation in response to various stimuli has been correlated with significant morphological changes [15]. Thus, blocking ERα phosphorylation at Ser216 aggravated microglia to increase both basal and LPS-induced activations. Brain extracts from adult ERα WT and KI mice treated with saline or LPS were subjected to RT-PCR analysis. LPS-induced mRNA levels of pro-inflammatory cytokines such as TNF-α, IL-1α and IL-1β were higher in ERα KI brains compared to ERα WT brains (Fig. 4a). Conversely, LPS-induced expression of an anti-inflammatory IL-10 mRNA slightly diminished LPS in ERα KI brains. In addition, LPS induction of mRNAs for two enzymes, Cox-2 and iNOS, were higher in ERα KI brains; in particular, iNOS mRNA was induced only in KI brains. Subsequently, cytokine arrays were utilized to examine expressions of cytokines at their protein levels (Fig. 4b). Only a few cytokines were detected in the extracts of saline-treated brains and there was no difference in these expressions between ERα WT and KI brains. LPS treatment induced numerous cytokines and chemokines in both ERα WT and KI brains (Fig. 4c). Semi-quantitation of these expressions revealed that the degrees of these inductions were much higher in ERα KI than in WT brain (Fig. 4c). As observed with their mRNAs, protein levels of IL-6, IL-1α and IL-1β also increased. However, no difference was observed in a TNF-α protein expression between WT and KI. In addition to cytokines, LPS-induced levels of chemokines and of a metalloprotease inhibitor TIMP-1 were higher in ERα KI than in WT brains. An integrin receptor ICAM-1 (also known as CD54) was also increased more in ERα KI brains after LPS treatment (Fig. 4b and c). These observations indicate that ERα KI microglia are more sensitive to LPS-elicited immune responses.

### Microglia in cultures

**Fig. 5:**
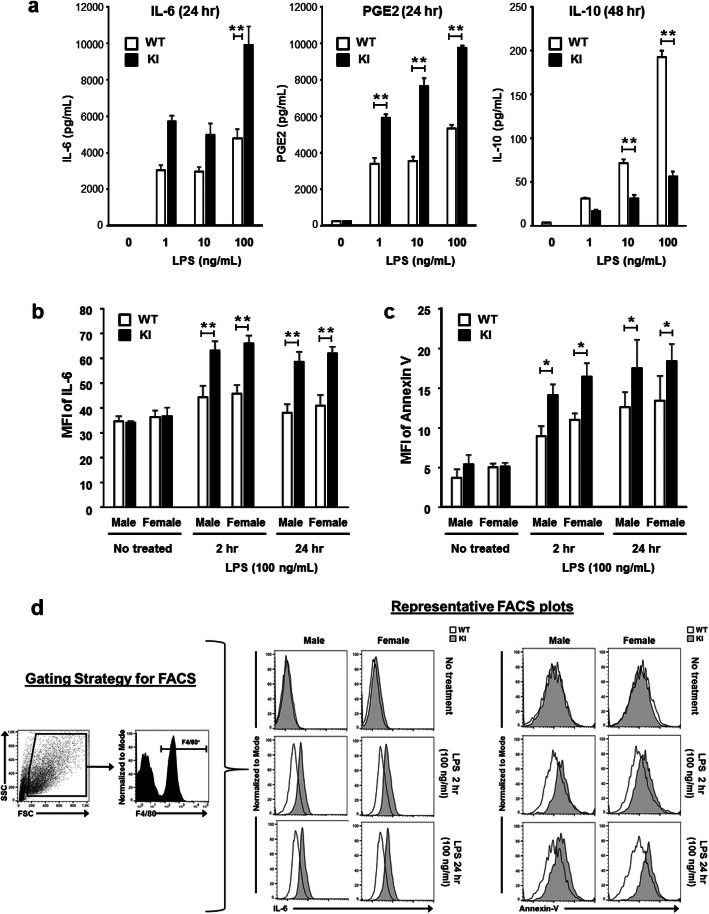
Expression of inflammatory factors in cultured microglia. **a** ELISA. Mixed glia cells were prepared from 5 neonates and cultured for 2 weeks and then treated with 1, 10 or 100 ng/mL of LPS or PBS for an additional 24 or 48 h. Media were recovered and subjected to ELISA for IL-6, PGE2 and IL-10. WT and KI denote ERα wild type and KI mice, respectively. ONE Way ANOVA plus post hoc test with Tukey-Kramer’s multiple comparisons test (Version 5.0, Stat view-j) was used for statistical analysis. Values are presented as means ± S.D.. **, p < 0.01, *n* = 4. **b** FACS. Mixed glia cells were separately prepared from male and female neonates, cultured for 2 weeks and then treated with 100 ng/mL of LPS or PBS for an additional 2 or 24 h. IL-6 production was determined by intracellular staining, followed by flow cytometry analysis. Assay was triplicated with three independent. ONE Way ANOVA plus post hoc test with Tukey-Kramer’s multiple comparisons test (Version 5.0, Stat view-j) was used for statistical analysis. Values are presented as means ± S.D.. **, *p* < 0.01. **c** Cell death was assessed by Annexin V staining, following by flow cytometry analysis. Assay was performed in triplicate eight times with three independent samples. These were separately prepared from male and female neonates. Two Way ANOVA plus post hoc test with Tukey-Kramer’s multiple comparisons test (GraphPad Prism) was used for statistical analysis. Values are presented as means ± S.D.. *, *p* < 0.05. **d** The gating strategy and the representative FACS plots for b and c

Mixed glia cells were prepared from brains of 2-day-old neonates, cultured for 2 weeks so that microglia matured, and treated with LPS, from which supernatants were collected for ELISA (Fig. 5a). The increases of pro-inflammatory factors IL-6 and PGE2 were about 50% higher in KI microglia after 24 h treatment. Conversely, an anti-inflammatory cytokine IL-10 was greatly repressed in KI microglia after 48 h treatment (Fig. 5a). Similar results were observed when examining intracellular levels of IL-6 by fluorescence-activated cell sorting (FACS). Briefly, mixed glia cells prepared from 2-day-old neonates were treated with PBS or LPS for 2 or 24 h. Male and female neonates were determined by different distances between anus and unitary opening. The mixed glial culture was the fixed and permeabilized to allow for antibody entry. Mixed glia cultures were stained for the microglia marker, F4/80, and F4/80^+^ microglia were analyzed for intracellular levels of IL-6. LPS-induced expression of IL-6 was approximately 35% higher in ERα KI than in ERα WT microglia (Fig. 5b). FACS was also utilized to analyze apoptosis in mixed glia cultures. Annexin V binds extracellular phosphatidylserine, which is actively exposed during apoptosis. After LPS treatment, we also observed about 30% increase in apoptosis in ERα KI microglia, as determined by Annexin V positivity (Fig. 5c). Noticeably, there are no sex differences in the responses. Taken together, these results suggested that ERα KI microglia became increasingly inflammatory as well as apoptotic after LPS treatment.

### Motor connection ability

**Fig. 6:**
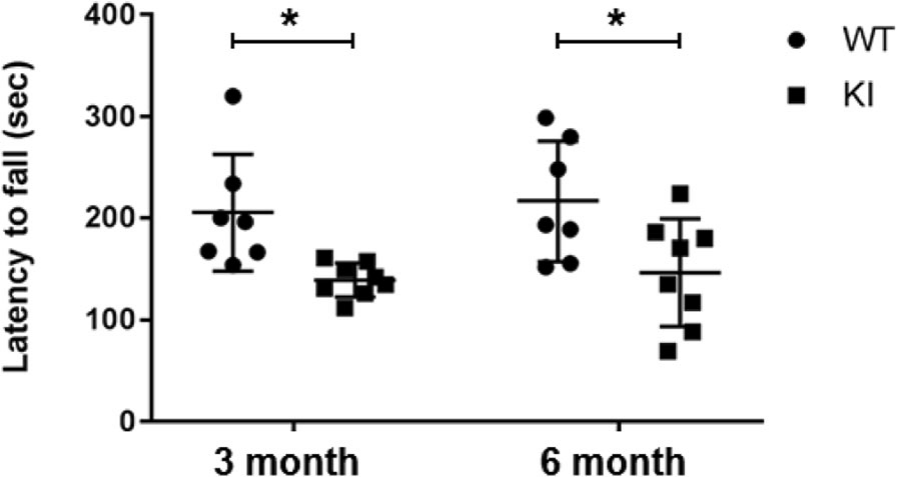
Latency to fall in rotarod test. Three-month-old and 6-month-old male mice were tested as described in the Experimental Procedure section. These values are represented as mean ± S.D. of 7–8 mice for each group. Data were analyzed by Two Way ANOVA plus post hoc test with Bonferroni’s multiple comparisons test (version 7.02, GraphPad Prism) and are shown as scatter plots. *, p < 0.05

Substantia nigra (SN) is an area of the brain that controls movement, microglia in which are associated with this function. Given the finding that inflammation was aggravated in microglia of ERα KI brains, Rotarod tests were employed to examine whether motor connection was affected. It was found that average latencies were 206 ± 57 s with 3-month old WT mice (Fig. 6). The corresponding KI mice significantly decreased this latency to 138 ± 17 s, impairing motor connection. These degrees of latency were continuously observed with 6-month old WT mice, 217 ± 59 and 146 ± 59 with WT and KI mice, respectively (Fig. 6). Thus, the microglia expression of ERα S216A mutant appeared to deteriorate motor connection of mice.

## Discussion

ERα is phosphorylated at Ser216 in microglia, only the immune cells in the brain. Microglia are the second immune cells which are found to express phosphorylated ERα. Neutrophils infiltrating the mouse uterus were first found to express phosphorylated ERα [10]. Studies with ERα S216A KI (*Esr1*^*S216A*^) mice show the anti-inflammatory and anti-apoptotic functions of phosphorylated ERα in microglia. Microglia in substantial nigra are activated in the brain of ERα S216A KI mice. Inflammatory activation of these microglia has been associated with neurodegenerative diseases such as Parkinson [16, 17]. ERα S216A KI mice and microglia can be excellent experimental models for us to further implicate phosphorylated ERα in inflammation-related neurodegenerative diseases and investigate their molecular and cellular mechanisms. ERα KO females developed obesity and decreased glucose tolerance, suggesting that ERα plays a crucial role in estrogen-mediated metabolic regulation [18]. ERα S216A KI mice also showed an obesity phenotype and an increase boll glucose levels in both males and females, indicating that phosphorylation is critical for ERα to regulate these phenotypes. However, unlike the case of ERα KO mice, this regulation may be both sex- and estrogen-independent.

Immunological study reported that M1/M2 polarization of macrophages plays an important role in controlling the balance between promotion and suppression in inflammation. Microglia protect brain cells from various stresses and insults by accelerating pro-inflammation to eliminate damaged cells (M1 microglia function) and, subsequently, decorrelating it to recover the brain form injury (M2 microglia function) [19]. Phosphorylated ERα is found to regulate both M1 and M2 functions, as indicated by the fact that an early expression of pro-inflammatory factors (e.g. IL-6 and PGE2) is stimulated and subsequent expression of anti-inflammatory factors (e.g. IL-10) is attenuated in LPS-treated ERα S216A KI mice. In other words, microglia in which ERα cannot be phosphorylated may have a strong function of M1 and may be less polarized to M2. Apoptosis is augmented in LPS-treated microglia from ERα S216A KI brains. In both the brain and cultured microglia, not all factors are regulated equally. However, phenotypes obtained by cultured microglia would reflect what should be observed in the brain. In addition, phosphorylated ERα may also have the potential to regulate microglia migration through the expression of cell adhesion molecules such as ICAM-1 and metalloprotease inhibitor TIMP-1. These patterns of regulations implicate phosphorylated ERα in the immune severance regulation to protect brains cells from injuries [20]. Rotarod tests found that ERα KI mice decreased motor ability. It remains to be seen if this correlate with decrease numbers of dopaminergic neurons in the areas of brain’s substantia nigra. In addition to neurodegenerative diseases, recently, macrophage-elicited PGE2-EP2-NF-κB signaling in brains was linked to chronic inflammation, cerebral aneurisms and subarachnoid hemorrhage with NF-κB suggested to be a therapeutic target of aneurisms [21]. However, phosphorylated ERα may be a better target, possibly controlling inflammation but not the other signals in the brain.

The stimulated inflammation observed with ERα S216A KI brains closely resembles previous reports that microglia are activated by LPS in global ERα KO mice [1], confirming the role of ERα in the inflammation. It was reported that estrogen repressed LPS-induced inflammation and apparently suppressed in isolated rat microglia from normal rat [22], suggesting the possibility that what this ERα-mediated regulation was an estrogen action. However, our repeated experiments with cultured microglia did not find any effects of estrogen on their activities (unpublished). These differences between the two experiments are not reconciled now and whether estrogen directly regulates phosphorylated ERα in microglia remains further investigated in the future. Functions of ERα are primarily understood by estrogen actions with a gender-dependent manner predominately observed in females [23–25]. An alternative is the possibility that estrogen indirectly regulates phosphorylated ERα through astrocytes. A cross-talk between astrocytes and microglia is well known to regulate inflammation in the brain [26, 27]. Moreover, a recent report find that microglia play the determining role in sexual differentiation of the brain and estrogen may regulate microglia through astrocytes [2, 28]. ERα is not phosphorylated in astrocytes (Fig. 1), possibly targeted by estrogen to transduce its signal to microglia.

Microglia are critically involved in the development of various neurodegenerative diseases including Alzheimer’s, Parkinson’s and Huntington’s diseases [16]. In fact, LPS-induced activation of microglia in the substantia nigra was associated with development of Parkinson disease [17, 29], although mice are known not to develop this disease [30]. Estrogen signaling has been investigated as a therapeutic target for developing drugs against neurotoxicity and injury as well as neurodegenerative diseases [26]. Phosphorylated ERα in microglia can be a more direct target of drug development. If chemicals that specifically bind and activate or inactivate phosphorylated ERα are identified, they may be effective in microglia but not the other cells in which ERα is not phosphorylated. Our previous study showed that human ERα S212D mutant activated ERE-reporter gene as observed with ERα WT and ERα S212A in Huh-7 cells [9], suggesting that cell-based reporter assays can be utilized to develop a high-throughput assay for identifying ligands specific to phosphorylated ERα, with cells such as Huh-7 and/or mouse microglia-derived BV-2 cells. In addition to the brain, other tissues such as skin (Langerhans cells in) and liver (Kupffer cells) house resident macrophages. The presence of ERα was previously reported in these resident macrophages [31, 32], as well as bone marrow-derived and peritoneal macrophages [33]. ERα S216A KI mice can be utilized to examine if their inflammatory responses are altered. In fact, Kupffer cells isolated from ERα S216A KI mice expressed cytokines differently from those from normal mice (unpublished). Once confirmed, anti-inflammatory drugs that target skin and/or liver may also be developed.

## Conclusions

ERα S216A KI mice are an excellent animal model for us to further investigate the inflammatory regulation of phosphorylated ERα and its molecular mechanism in microglia and implicate microglia in inflammation-related brain diseases. Current findings with this mouse provide experimental bases for us to further extend functional study of phosphorylated ERα into various directions which include generation of microglia-specific aberration of Ser216 phosphorylation. Once ERα is confirmed in human microglia, therapeutic drugs which selectively target phosphorylated ERα over non-phosphorylated ERα. Together with our investigations using ERα S216A KI mice and RXRα T167A KI mice [8], we demonstrated the possibility that phosphorylation of the conserved motif within the DBD can be a common signal for nuclear receptors to diverge their functions.

## Methods

### Materials

An antibody against an anti-phospho-S216 peptide of ERα (αP-S216) was produced and evaluated by AnaSpec Inc. (San Jose, CA). Iba-1 antibody and Antibody Diluent were purchased from WAKO; biotinylated goat anti-rabbit antibody and Vectastain ABC reagents from Vector Laboratory (Burlingame, CA); Mouse Cytokine Array Panel A and an anti-F4/80 antibody from R&D Systems, Inc.; an antibody against green fluorescent protein (HRP-conjugated) from Abcam; an anti-Iba-1 antibody from Gene Tex; an Alexa 594 anti-Rat antibody and DMEM/F12 media from Life Technologies; Fixation/Permeabilization Solution Kit (BD Biosciences, San Jose, CA). Trizol from Life Technologies; Direct-zol™ RNA kit from Zymo Research; RNeasy mini kit from Qiagen; MultiScribe Reverse Transcriptase from Applied Biosystems; an anti-GFAP antibody from STEMCELL Technologies; Precision Plus Protein Standards from Bio-Rad. All reagents are highest qualities commercially available.

Animals: Mice were maintained on a 12 h light/12 h dark cycle and fed with NIH-31 the Open Formula Autoclavable diet (Zeigler, PA) and water ad libitum. Ex3-ERα KO mice were generous gift from Dr. Korach’ lab. All research has been reviewed and approved by an Institutional Animal Care and Use Committee of NIEHS/NIH. LPS solution (1 mg/ml) was kept at − 20 °C and thawed just before intraperitoneal injection. All experiments were performed in accordance with relevant guidelines and regulations.

### Generation ERα S216A KI (*Esr1*^*S216A*^) mice

A 5.1-kb left arm containing introns 2 and 3 and exon 3 and a 2.0-kb right arm containing intron 3 were amplified from genomic DNAs of C57B/6 were cloned into pCR-XL-TOPO (Thermo Fisher Scientific, MA). Codon serine 216 encoded by exon 3 in the left arm was changed to alanine by site directed mutagenesis. After digestion with restriction enzymes, these DNA arms were cloned into the targeting vector which carries two multi cloning sites, self-excising ACN cassette [34] and DT-A cassette. The ACN cassette contains a testis-specific promoter from the angiotensin-converting enzyme gene that drives the expression of the Cre-recombinase gene, and RNA pol II promoter was used to drive neomycin registrant (neor) gene as a selection marker, which allows to screen ES cells in the presence of G418. When chimeras those are born from theses targeted ES-cells containing the ACN cassette are bred for germline transmission, somatic cells derived from the ES cells retained the cassette, but self-excision occurred in all ES-cell-derived sperm, and as a result, the unexpected consequence due to the presence of Neo gene in chromosome can be avoided all together. The linearized targeting vector was electroporated into G4 embryonic stem cells (B6129F1 genetic background). The G418-resistant ES clones were screened by Southern blot analysis of KpnI- and BglI- digested genomic DNA with 5′ and 3′ external probes. BglI-digestion generated 12- and 8.3-kb bands for KI and WT, respectively, detected by using the 3′-genomic probes. These Southern probes were amplified from ES cell genomic DNA: 5′ probe, 130966/131442 (tgcagctgcttcctactggcttgaatcatccataagatattaataagcaaacagtaaaaagatctgcggttggtaggggagttcaatactatgatgatgaaatggaaagtgatgggtaatagaataggaacaagaactggaagctttgagccaatgctctctaaggatcactaaaaagtaaagaaatcctatctgaggctgccagcctcagagctaagttatttagagtggaaaaagttggccaactcagatggactcaaaccaaggagccaatgttttgtgagttttatagccgatgtcatttacgaaccattaaaatattgtattcaatattaatgagggggaatagcagggaagggttatgaaatacgagctgaaaggaaagctaggcctttgaggggaggaccagtgagttcatgtggctttgctatttgggacatggtgggactatgaagcagggaaccaggagcttcctta; 3′ probe, 139971/140460 (ctgaagagatggctcagtggttaagagcatccactgtttgctcttccagaggattctggttcaattcccagctctcacatagcagttaataactgtctgtaaactccagttcaagaggacctggcaccctcacacagacatacatgcaggcaaaacaccaatgcacataaaaataaatacatagtttaagaacttcaggctcacttagcaggctctgtgtacttgtagagatctgtttctttaatcttggggactcacctctgtccactcaagaggggctgtgctcacacttcttttcaaattagttttctctctctgagtgcattcatgtgaagagtaggaggaatactagaagagccacccatttcttcacaggattattttatttctgagatctttttagagatatgtctgatcctatccactcccagcaaatagtaagtctttgttctcaacatttccactcatgaccctctctagtctgtaacag). Correctly targeted ES clones were microinjected into albino C57BL/6 J blastocyst and non-surgically transferred to pseudo-pregnant SWISS Webster females. Male germline chimeric founders were bred to wildtype C57BL/6 J females, which resulted in the removal of the self-excising positive selection marker. Self-excision of the positive selection cassette was confirmed by conventional PCR. Following removal of the positive selection marker, the line was crossed to C57BL/6 J wildtype mice one more generation, prior to in-crossing, and the resulting to obtain knock-in mice. Primers for genotyping by PCR used for 5′ probe 5′-TGCAGCTGCTTCCTACTGGCTTGA-3′ and 5′-TAAGGAAGCTCCTGGTTCCCTGCT-3′; for 3′ probe, 5′-CTGAAGAGATGGCTCAGTGGTTAA − 3′ and 5′-CTGTTACAGACTAGAGAGGGT-3′.

### Primary cortical mixed glial culture

Primary cortical mixed glial cultures were prepared from brains of mice at postnatal day 1–3, as previously described [35, 36]. Cortices were isolated from brains from which meninges and blood vessels were removed using forceps. Cells were dispersed by dissociating tissues in DMEM/F12 media through trituration. Obtained cell suspension were plated on either 24-well plates or 96-well plates pre-coated in poly-D-lysine (20 μg/ml) at 1 × 10^5^ cells/well or 5 × 10^4^ cells/well. Cells were maintained in DMEM-F12 (1:1) media supplemented with 10% heat-inactivated fetal bovine serum, 2 mM L-glutamine, 1 mM sodium pyruvate, 100 μM non-essential amino acids, 50 U/ml penicillin, and 50 μg/ml streptomycin. Culture medium was changed every 3 days. To allow high yield of microglia in the culture, the cells were cultured for 2 weeks. The mixed glial cultures with a ratio of 20% microglia and 80% astrocytes were obtained [35].

### Microglia-enriched cultures

Mouse microglia-enriched cultures were prepared from primary mixed glial cultures as previously described (1, 2). Mixed glia cultures were plated on 150 cm^3^ flasks pre-coated with poly-D-lysine (20 μg/ml) at 5 × 10^7^ cells/flask and maintained in DMEM-F12 media changed every 3 days for 2 weeks. Then, matured microglia were shaken off at 180 rpm for 40 min and re-plated on glass-bottom culture dishes (MatTek, Ashland, MA, USA) pre-coated with poly-D-lysine (20 μg/ml) at 1 × 10^6^ cells/well.

### Immunohistochemical staining

Mouse brain was first perfused with cold PBS to remove bloods, from which sections (35 μm thick) were prepared. Brain sections were treated with 1% hydrogen peroxide for 10 min, incubated for 20 min with blocking solution (BSA 1%/Triton X-100 0.4%/Normal Goat Serum 4% in PBS) and incubated overnight at 4 °C with rabbit polyclonal antibody against Iba-1 (1:4000) in Antibody Diluent. Stained sections were washed in PBS three times each for 10 min and incubated for 2 h with PBS containing 0.3% Triton X-100 and a biotinylated goat anti-rabbit antibody (1:227). After washing three times with PBS, these sections were incubated for 1 h with the Vectastain ABC reagents diluted in PBS containing 0.3% Triton X-100. Finally, these treated sections were incubated with 3, 3′-diaminobenzidine and urea-hydrogen peroxide tablets dissolved in water to visualize microglia. The nigral densities of the Iba-1 immunostaining were measured using ImageJ software. To quantify the Iba-1 staining of microglial cells in the substantia nigra, representative images of Iba-1-positive regions in the substantia nigra were captured at 40× magnification. A total of 100 microglia in each mouse were selected randomly, and the Iba-1 density was measured and normalize with size of area selected. One way ANOVA plus post hoc test with Bonferroni’s multiple comparisons test was used to analyze the difference between saline injected WT microglia vs. LPS-injected WT or LPS-injected ERα KI.

### Elisa

Cells were harvested and centrifuged to collected cultured media at time points after LPS (Millipore) treatment. Cytokine and metabolite concentrations were measured by ELISA. IL-6, IL-10 and PGE2 ELISA kits purchased from R&D Systems. ELISA assays were performed according to the manufacturer’s instructions.

Cytokine antibody array: Mouse brains were homogenized in 500 μL of cold PBS containing protease inhibitor cocktail and 5 μL of Triton-X100 and centrifuged at 10,000×g for 5 min at 4 °C. Obtained lysates (300 μg) were subjected to cytokine protein array. Cytokine expressions were detected by a mouse cytokine antibody array, panel A kit according to the manufacturer’s instructions. Obtained spots were measured as densities by ImageJ software and showed in a graph. Cytokine mouse antibody array were performed according to the manufacturer’s instructions.

### Double immunofluorescence staining

Immunofluorescence staining was performed as previously described [37]. Mouse mixed glia or enriched microglia cells were cultured on 35 mm bottom glass dishes, fixed with 4% formaldehyde and blocked with a goat normal serum in PBS buffer for 20 min. For the first staining, these dishes were incubated with given antibodies such as an anti-ERα and P-S216 antibody for 30 min at room temperature. For the second staining, stained dishes were washed with PBS buffer and incubated with marker antibodies such as anti-Iba-1 and GFAP antibodies for 30 min at room temperature. Subsequently, after washed with PBS, these dishes were incubated with a goat anti-rabbit IgG secondary antibody, Alexa Fluor 488 and a goat anti-mouse IgG secondary antibody, Alexa 594 (1:500) (Thermo Fisher) mixture at room temperature for 1 h in the dark. These stained cells were washed with PBS buffer and mounted with mounting medium containing DAPI (VECTASHIELD®). Stained cells in glass bottom dishes were observed using Zeiss 710 confocal microscopy (Zeiss).

### Flow Cytometry

To assess cell death, mixed glia cultures were stained with an anti-F4/80 antibody (1:50) for 30 min on ice and Alexa 594 anti-rat antibody (1:500), followed by staining by Annexin-V as previously described [38], and acquisition on flow cytometer (LSRII, BD Bioscience). Annexin-V positivity was analyzed for F4/80^+^ microglia singlets from mixed glia cultures using FlowJo software. To asses IL-6 expression, mixed glia cells were stained with an anti-F4/80 antibody (1:50) for 30 min on ice and Alexa 594 anti-rat antibody (1:500), followed by fixation/permeabilization using Fixation/Permeabilization kit (BD PharmingenTM), washed in FACS buffer (PBS, 2% BSA, 0.1 mM EDTA, 0.1% sodium azide), collected by centrifugation and re-suspended in BD Perm/WashTM buffer. Then these cells were incubated with anti-PE-IL-6 antibody for 20 min on ice in dark. After washing, stained cells were suspended in 200 μL of FACS buffer for immediate acquisition on flow cytometer (LSRII, BD Bioscience). The mean fluorescence intensity (MFI) for IL-6 in F4/80^+^ microglia singlets from mixed glia cultures was calculated using FlowJo software.

### Western blots

Mouse uteri or brains were homogenized in 50 mM Tris-HCl buffer saline (pH 7.6) containing 8 M urea and 1% SDS. After centrifugation, resulting supernatant was added to SDS sample buffer. Protein concentrations were determined by Bio-Rad protein assay (Bio-RAD, Hercules, CA). Proteins were separated on a SDS-PAGE and transferred onto PVDF membranes (GE Healthcare, Pittsburgh, PA). These membranes were blocked with 5% BSA or 5% skim milk in 50 mM Tris-HCl-buffered saline containing 0.1% Tween-20 (TBS-T), incubated with given primary antibodies, washed with TBS-T, incubated with HRP-conjugated secondary antibodies and visualized using WesternBright Sirius HRP substrate (Advansta, Menlo Park, CA).

### RT-PCR

Total RNAs were extracted from of enrich microglial cells using Trizol and a Direct-zolTM RNA kit. An RNeasy mini kit was used to extracts RNAs from mouse brains according to these manufacturer’s instructions cDNAs were synthesized using MultiScribe Reverse Transcriptase. Real-time PCR was performed using an ABI prism 7700 sequence detection systems (Applied Biosystems) with following TaqMan probes (Applied Biosystems) used: human and mouse glyceraldehyde-3-phosphate dehydrogenase (Hs99999905_m1 and Mm99999915_g1) for an internal control, IL-1α (Mm00434228_m1), IL-1β (Mm00434228_m1), IL-6 (Mm00446190_m1), IL-10 (Mm00439614_m1) and iNOS (Mm00440485-m1). Primers used for Cox-2 were primer-L: CAAGACAGATCATAAGCGAGGA and –R: GGCGCAGTTTATGTTGTCTGT. Assays were performed with a 7900HT Fast Real-Time PCR System (Applied Biosystems).

### Rotarod test

Rotarod test was conducted by Rotamex-5 (Columbus instruments, Columbus, OH, USA). Groups of 7 ERα WT and of 8 ERα KI males were trained for 4 consecutive days before they were tested at their 3- and 6-month of ages. Initial rotation of rotarod was set at 1 rpm and incrementally accelerated 1 rpm every 12 s. Retention times (latencies) on rotarod to fall off from the rotarod was measured three times for each mouse and averaged.

### Statistical analysis

Statistical analyses were conducted with One- or Two -Way ANOVA plus post hoc test with Bonferroni’s multiple comparisons test or Tukey-Kramer’s multiple comparisons test. Values are presented as means ± S.E. or ± S.D.

## Supplementary information

**Additional file 1: Figure S1.** Amino acid sequence alignments of mouse nuclear receptors. Amino acid sequence alignments of mouse nuclear receptors to show the conserved phosphorylation site within the DNA binding domains.

## Data Availability

Not applicable.

## Abbreviations

Cox-2: Cyclooxygenase-2
ELISA: Enzyme linked immunosorbent assay
ERα: Estrogen receptor α
FACS: Fluorescence-activated cell sorting
ICAM-1: Intercellular adhesion molecule-1
MFI: Mean fluorescence intensity
IL: Interleukin
iNOS: inducible nitric oxide synthase
KI: Knock-in
KO: Knockout
LPS: Lipopolysaccharide
NF-κB: Nuclear factor-kappa B
PCR: Polymerase chain reaction
PGE2: Prostaglandin E2
RXRα: Retinoid X receptor α
TIMP-1: Tissue inhibitors of metalloproteinases-1
TNFα: Tumor necrosis factor α
WT: Wild type
