# Supplementary material for: Estrogen receptor α phosphorylated at Ser216 confers inflammatory function to mouse microglia
Source: Cell Commun Signal. 2020 Jul 29;18:117. doi: 10.1186/s12964-020-00578-x (PMC7390202; doi:10.1186/s12964-020-00578-x)
Supplement: Supplementary file 2 — Additional file 1: Figure S1. Amino acid sequence alignments of mouse nuclear receptors. Amino acid sequence alignments of mouse nuclear receptors to show the conserved phosphorylation site within the DNA binding domains. [file 12964_2020_578_MOESM2_ESM.pdf]

Additional file 1 information

Estrogen receptor α phosphorylated at Ser216 confers inflammatory function to mouse microglia

Sawako Shindo, Shih-Heng Chen, Saki Gotoh, Kosuke Yokobori, Hao Hu, Manas Ray, Rick Moore, Kiyoshi Nagata, Jennifer Martinez, Jau-Shyong Hong, Masahiko Negishi

Additional file 1: Figure S1. Amino acid sequence alignments of mouse nuclear receptors. Amino acid sequence alignments of mouse nuclear receptors to show the conserved phosphorylation site within the DNA binding domains.

NR1A1	(TRα)	71	EGCKGFFRRTIQKNLHPTYSCKY	93
NR1A2	(TRβ)	139	EGCKGFFRRTIQKSLHPSYSCKY	161
NR1B1	(RARα)	106	EGCKGFFRRSIQKNMVYTCHRDK	128
NR1B2	(RARβ)	133	EGCKGFFRRSIQKNMIYTCHRDK	155
NR1B3	(RARγ)	108	EGCKGFFRRSIQKNMVYTCHRDK	130
NR1C1	(PPARα)	120	EGCKGFFRRTIRLKLVYDKCDRS	142
NR1C2	(PPARδ)	91	EGCKGFFRRTIRMKLEYEKCDRI	113
NR1C3	(PPARγ)	157	EGCKGFFRRTIRLKLIYDRCDLN	179
NR1D1	(Rev-erbα)	151	EGCKGFFRRSIQQNIQYKRCLKN	173
NR1D2	(Rev-erbβ)	121	EGCKGFFRRSIQQNIQYKKCLKN	143
NR1F1	(RORα)	91	EGCKGFFRRSQQSNATYSCPRQK	113
NR1F2	(RORβ)	39	EGCKGFFRRSQQNNASYSCPRQR	61
NR1F3	(RORγ)	49	EGCKGFFRRSQQCNVAYSCTRQQ	71
NR1H2	(LXRβ)	96	EGCKGFFRRSVVHGGAGRYACRG	118
NR1H3	(LXRα)	114	EGCKGFFRRSVIKGARYVCHSGG	136
NR1H4	(FXRα)	156	EGCKGFFRRSITKNAVYKCKNGG	178
NR1H5	(FXRβ)	140	EGCKGFFRRSITKNAVYSCKNGG	162
NR1I1	(VDR)	42	EGCKGFFRRSMKRKALFTCPFNG	64
NR1I3	(CAR)	39	EGCKGFFRRTVSKTIGPICPFAG	61
NR2A1	(HNF4α)	78	DGCKGFFRRSVRKNHMYSCRFSR	100
NR2A2	(HNF4γ)	40	DGCKGFFRRSIRKSHVYSCRFSR	62
NR2B1	(RXRα)	158	EGCKGFFKRTVRKDLTYTCRDNK	180
NR2B2	(RXRβ)	210	EGCKGFFKRTIRKDLTYSCRDNK	232
NR2B3	(RXRγ)	157	EGCKGFFKRTIRKDLIYTCRDNK	179
NR2C1	(TR2)	119	EGCKGFFKRSIRKNLVYSCRGSK	141
NR2C2	(TR4)	168	EGCKGFFKRSVRKNLTYSCRSSQ	190
NR2E1	(TLX)	34	DGCSGFFKRSIRRNRTYVCKSGN	56
NR2E3	(PNR)	58	NGCSGFFKRSVRRRLIYRCQVGA	80
NR2F1	(COUP-TFI)	101	EGCKSFFKRSVRRNLTYTCRANR	123
NR2F2	(COUP-TFII)	97	EGCKSFFKRSVRRNLSYTCRANR	119
NR2F6	(COUP-TFIII)	75	EGCKSFFKRSIRRNLSYTCRSNR	97
NR3A1	(ERα)	207	EGCKAFFKRSIQGHNDYMCPATN	229
NR3A2	(ERβ)	186	EGCKAFFKRSIQGHNDYICPATN	208
NR3B1	(ERRα)	97	EACKAFFKRTIQGSIEYSCPASN	119
NR3B2	(ERRβ)	142	EACKAFFKRTIQGNIEYNCPATN	164
NR3B3	(ERRγ)	146	EACKAFFKRTIQGNIEYSCPATN	168
NR4A1	(NGFIB)	288	EGCKGFFKRTVQKSAKYICLANK	310
NR4A2	(NURR1)	281	EGCKGFFKRTVQKNAKYVCLANK	303
NR4A3	(NOR1)	311	EGCKGFFKRTVQKNAKYVCLANK	333
NR5A1	(SF1)	31	ESCKGFFKRTVQNNKHYTCTESQ	53
NR5A2	(LRH1)	125	ESCKGFFKRTVQNQKRYTCIENQ	147
NR6A1	(GCNF)	93	EGCKGFFKRSICNKRVYRCSRDK	115
NR1I2	(PXR)	56	EGCKGFFRRAMKRNVRLRCPFRK	78
NR3C1	(GR)	455	GSCKVFFKRAVEGQHNYLCAGRN	477
NR3C2	(MR)	621	GSCKVFFKRAVEGQHNYLCAGRN	643
NR3C3	(PR)	578	GSCKVFFKRAMEGQHNYLCAGRN	600
NR3C4	(AR)	557	GSCKVFFKRAAEGKQKYLCASRN	579
NR0B1	(DAX-1)			
NR0B2	(SHP)			
